# Prognostic Value of Circulating Tumour DNA in Asian Patients with Hepatocellular Carcinoma: A Systematic Review and Meta-Analysis

**DOI:** 10.1155/2022/8019652

**Published:** 2022-02-24

**Authors:** Hongli Liu, Hong Yang, Xiaoliang Chen

**Affiliations:** Chongqing University Cancer Hospital, Chongqing 400030, China

## Abstract

**Background:**

Circulating tumour DNA (ctDNA) is a noninvasive method of detecting tumours, and its prognostic significance in hepatocellular carcinoma (HCC) patients is controversial. We conducted a systematic review of published research data to evaluate the prognostic value of ctDNA in HCC patients.

**Methods:**

The PubMed, Embase, Web of Science, Cochrane Library, and Scopus databases were searched to identify eligible studies reporting disease-free survival (DFS) and overall survival (OS) stratified by ctDNA prior to January 2022. We evaluated the quality and design of these studies. The hazard ratio (HR) was used to combine the survivorship curve and univariate and multivariate results of the included studies.

**Results:**

In total, 8 articles were included, encompassing 577 HCC patients. The results of survival curve analysis showed that ctDNA was related to poor OS and DFS, and the effect sizes were HR = 2.44, 95% CI (1.42, 4.20), *P*=0.001; HR = 2.63, 95% CI (1.96, 3.53), *P* < 0.001. The univariate analysis results showed that ctDNA was related to poor OS (HR = 4.48, 95% CI (1.17, 13.70), *P*=0.003). The combined results of multivariate analysis showed that ctDNA was related to a shorter risk of OS (HR = 3.74, 95% CI (1.45, 9.65), *P*=0.006). The univariate and multivariate descriptive analysis results showed that ctDNA was related to shorter DFS, and the effect sizes were HR = 3.28, 95% CI (1.23, 11.30), *P*=0.011; HR = 3.01, 95% CI (1.11, 10.5), *P* < 0.001.

**Conclusion:**

The evidence provided by this analysis suggests that ctDNA may be a prognostic biomarker and is negatively correlated with the survival of HCC patients. Mutations in the TERT and SOCS3 promoters in ctDNA are associated with poor prognosis and are expected to become good targets for liquid biopsy and to help select treatment strategies.

## 1. Introduction

Hepatocellular carcinoma (HCC) is one of the most common malignant tumours worldwide. Because the early clinical manifestations of HCC patients are not clear, the symptoms are hidden, and there is a lack of specific and sensitive early diagnostics, most patients are in the middle and late stages of diagnosis and miss the opportunity for radical resection. In addition, microvascular invasion and distant metastasis can occur in the early stage of HCC, and the postoperative recurrence and metastasis rates of patients are extremely high. According to statistics, the 5-year recurrence rate after HCC resection is as high as 70%, and blood metastasis is the most common [1, 2]. Therefore, there is an urgent need for specific and sensitive serological markers for effective early detection and postoperative recurrence monitoring, as well as effective measures to prevent HCC metastasis and recurrence [3–5].

Recent studies have found that systemic inflammatory responses are closely associated with tumour development, such as the neutrophil-to-lymphocyte ratio, lymphocyte-to-monocyte ratio, and platelet-to-lymphocyte ratio, which have been shown to correlate with the prognosis of a variety of malignancies [6–10]. However, the heterogeneity of serum inflammatory markers can make their applicability questionable. With the development of genetic technology, scholars have attempted to predict the occurrence of disease and prognostic assessment from a genetic perspective. Tumour cells can release nucleic acid fragments into the circulation, which become circulating tumour DNA (ctDNA), and this may be considered a new generation of tumour molecular markers [11, 12]. ctDNA carries the gene mutation information of all tumour cells, which can be detected in the early stages of tumour development. Therefore, tumour “liquid biopsy” based on ctDNA detection can improve noninvasive, real-time, and comprehensive dynamic monitoring of tumours while overcoming the limitations of traditional tissue puncture [13, 14]. Similar to most malignant tumours, the development of liver cancer is accompanied by genetic and epigenetic mutations [15]. ctDNA can be detected in the peripheral blood of HCC patients and carries genomic information of the HCC tumour tissue. The quantitative level of ctDNA, methylation detection, copy number variation, and gene mutation are closely related to HCC, and the analysis of ctDNA can help diagnose HCC, guide antitumour drug selection, and predict prognosis.

Among the many possible applications, the prognostic and predictive value of ctDNA in HCC has attracted the most intense interest [16, 17]. Studies have found that ctDNA may be a reliable prognostic factor with poor outcomes [18–20]. A positive test for ctDNA means that HCC patients undergoing surgery, chemotherapy, or targeted therapy are at higher risk of recurrence or short overall survival (OS) [21–23]. However, other studies have found no differences in survival rates between tDNA-positive and ctDNA-negative patients [24, 25]. To clarify the prognostic role of ctDNA in HCC, we conducted a systematic literature review to better understand its prognostic value in HCC patients.

## 2. Methods

### 2.1. Criteria for Inclusion

(1) The patient was clearly diagnosed with liver cancer by pathology; (2) ctDNA was separated and obtained from serum, plasma, or peripheral blood; (3) hazard ratio (HR) and 95% confidence interval (CI) could be obtained directly or indirectly; (4) when multiple articles reported on the same population, the latest literature was included; and (5) belonged to a cohort study.

### 2.2. Criteria for Exclusion

(1) Lack of survival data; (2) failure to obtain the full text; and (3) reviews, letters, case reports, and conference abstracts that did not match the type of literature.

### 2.3. Search Strategy

This research searched PubMed, Embase, Web of Science, and Cochrane Library. The search start and end times were from the establishment of the database to January 2022. The search terms included “liver cancer, liver tumour, circulating DNA, plasma DNA, serum DNA, and prognosis.” A combination of subject words and free words was used to search. First, irrelevant documents were excluded by reading the title, author information, and abstract of the document. Second, repetitive documents, non-Chinese and English documents, reviews or correspondence documents, and other inconsistent document types were excluded. Finally, the full text was read, and documents that met the inclusion criteria were included in the study.

### 2.4. Data Extraction

Two researchers independently extracted the following basic data from the included literature: the name of the first author, the year of publication, the country of publication, the clinical stage, the sample size, the source of the specimen, the detection method, the type of ctDNA markers, and the endpoint event. In this meta-analysis, the HR of the endpoint event and its 95% CI value were used as the combined effect size. Then, it was necessary to obtain the HR and its 95% CI value of each article. The HR of the endpoint event and its 95% CI were not directly reported in the literature. We HRs and 95% CIs were obtained from the Kaplan–Meier survival curves using Engauge Digitizer version 4.1 (http://digitizer.sourceforge.net/).

### 2.5. Quality Assessment

The literature is a cohort study, and the Newcastle–Ottawa Scale (NOS) was used to evaluate the quality of the literature [26, 27]. NOS is mainly scored from three aspects: the selection, comparability, and outcome of the study subjects, including the following 8 scoring items: (1) how representative is the cohort study of the exposure group; (2) the selection of the cohort study of the nonexposure group; (3) determination of exposure factors; (4) no outcome event occurred at the beginning of the study; (5) the study controlled for the most important and other confounding factors; (6) evaluation of outcome events; (7) observed the outcome events and whether the follow-up was sufficient; and (8) whether the follow-up was complete. Except for Item 5, which was 2 points, all others were 1 point. The full NOS score was 9 points, and the total score was less than 5 points, which was regarded as low-quality literature and was excluded. The two researchers independently completed the above scoring; if they disagreed, the findings were submitted to a third researcher for negotiation.

### 2.6. Statistical Analysis

Stata 14.0 software (STATA Corporation, College Station, TX, USA) was used for statistical analysis. HR was used to evaluate the relationship between ctDNA and the prognosis of liver cancer, where HR > 1 indicates a poor prognosis. We merged them separately according to the source of survival data (survivorship curve, univariate and multivariate). Before merging the HR and its 95% CI, the heterogeneity between the literature was first tested. The *Q* test and I^2^ test were used to evaluate the heterogeneity, and the *I*^2^ value represents the degree of heterogeneity. When the *P* value > 0.1 or *I*^2^ value < 50%, there was no obvious heterogeneity, and the fixed effects model was selected to combine HR and its 95% CI. Otherwise, it suggested obvious heterogeneity, and the random effects model was selected to combine HR and its 95% CI. Publication bias was evaluated through Egger's and Begg's tests. Some potential factors may have affected the prognostic evaluation of breast cancer by ctDNA; thus, it was necessary to conduct subgroup analysis. Sensitivity analysis adopts a one-by-one elimination method.

## 3. Results

**Figure 1:**
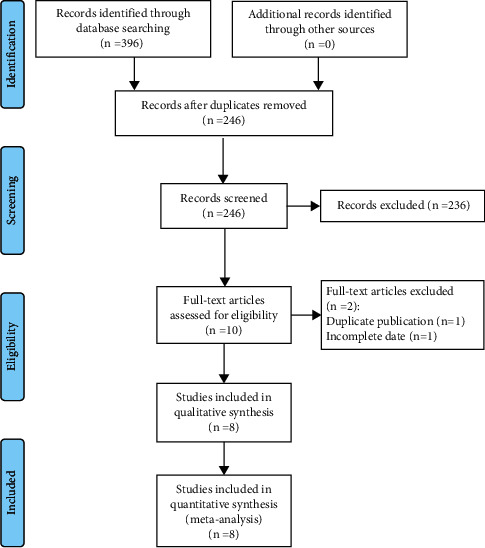
Flow diagram showing literature filtration process.

According to the formulated search formula, 396 articles were retrieved from the PubMed, Embase, Web of Science, and Cochrane Library databases. By reading the title and abstract, 236 inconsistent documents were excluded. Finally, the full text of the remaining 10 documents was read, and 2 inconsistent documents were excluded according to the inclusion and exclusion criteria. Finally, 8 articles [21–25, 28–30] that complied with the literature were included in the meta-analysis. The detailed literature screening process is shown in Figure 1.

### 3.1. Basic Features of the Included Literature

According to the scores of the NOS scale, all of the studies scored 7 points or above, and there were no low-quality studies. Eight studies were published from 2006 to 2020, including a total of 577 HCC patients. These studies were from the Asian region. The basic characteristics of the included studies are shown in Table 1.

### 3.2. Impact of ctDNA on the OS of HCC

**Figure 2:**
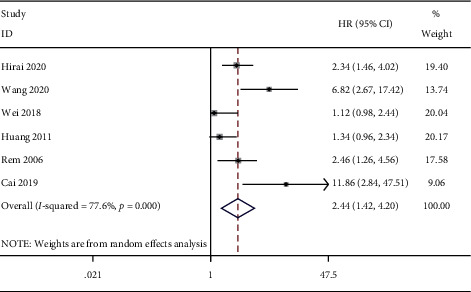
Forest plots of the pooled hazard ratio (HR) for the impact of ctDNA on survivorship curve OS.

**Figure 3:**
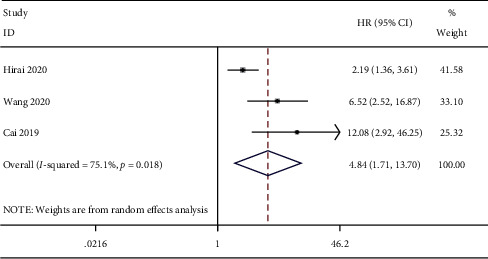
Forest plots of the pooled hazard ratio (HR) for the impact of ctDNA on univariate OS.

**Figure 4:**
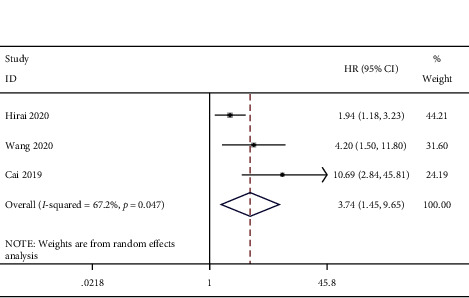
Forest plots of the pooled hazard ratio (HR) for the impact of ctDNA on multivariate OS.

We conducted a combined analysis based on the source of the survival data. The results of survival curve analysis showed that ctDNA was related to poor OS (HR = 2.44, 95% CI (1.42, 4.20), *P*=0.001) (Figure 2). Univariate analysis also showed that ctDNA was related to poor OS (HR = 4.48, 95% CI (1.17, 13.70), *P*=0.003) (Figure 3). The combined results of multivariate analysis showed that ctDNA was related to a shorter risk of OS (HR = 3.74, 95% CI (1.45, 9.65), *P*=0.006) (Figure 4).

### 3.3. Impact of ctDNA on the DFS of HCC

**Figure 5:**
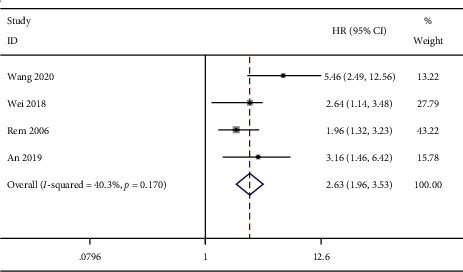
Forest plots of the pooled hazard ratio (HR) for the impact of ctDNA on survivorship curve DFS.

Survival curve analysis showed that ctDNA was related to poor DFS (HR = 2.63, 95% CI (1.96, 3.53), *P* < 0.001) (Figure 5). The descriptive analysis results for univariate analysis and multivariate analysis showed that ctDNA was associated with shorter DFS (univariate: HR = 3.28, 95% CI (1.23, 11.30), *P*=0.011; multivariate: HR = 3.01, 95% CI (1.11, 10.5), *P* < 0.001).

### 3.4. Subgroup Analysis

Subgroup analysis was conducted according to the detection method and the type of markers. The OS, ddPCR, and other detection methods derived from the survival curve were related to OS, and the combined effect sizes were HR = 3.70, 95% CI (1.31, 10.45), *P*=0.013; HR = 1.97, 95% CI (1.05, 3.70), *P*=0.035. The markers TERT and ctDNA were related to OS, and the combined effect sizes were HR = 2.34, 95% CI (1.41, 3.88), *P*=0.001 and HR = 3.46, 95% CI (1.43, 8.34), *P*=0.006, respectively. However, the marker SOCS3 was not related to OS, and the combined effect sizes were HR = 1.12, 95% CI (0.71, 1.75), *P*=0.626.

For OS from univariate analysis, ddPCR and other detection methods were related to OS, and the combined effect sizes were HR = 3.49, 95% CI (1.21, 10.05), *P*=0.021; HR = 12.08, 95% CI (3.04, 48.08), *P* < 0.001. The markers TERT and ctDNA were related to OS, and the combined effect sizes were HR = 2.19, 95% CI (1.34, 3.57), *P*=0.002; HR = 7.95, 95% CI (3.63, 17.40), *P* < 0.001.

For OS from multivariate analysis, ddPCR and other detection methods were related to OS, and the combined effect sizes were HR = 2.49, 95% CI (1.23, 5.06), *P* < 0.001; HR = 10.69, 95% CI (2.84, 45.81), *P*=0.001. The markers TERT and ctDNA were related to OS, and the combined effect sizes were HR = 1.94, 95% CI (1.17, 3.21), *P*=0.01; HR = 5.94, 95% CI (2.45, 14.37), *P* < 0.001.

For DFS, ddPCR and other detection methods derived from the survival curve were related to DFS, and the combined effect sizes were HR = 5.46, 95% CI (2.49, 12.56), *P* < 0.001; HR = 2.35, 95% CI (1.71, 3.22), *P* < 0.001. The markers ctDNA and SOCS3 were related to DFS, and the combined effect sizes were HR = 2.62, 95% CI (1.86, 3.71), *P* < 0.001; HR = 2.64, 95% CI (1.51, 4.61), *P*=0.001. The results of the overall and subgroup analyses are shown in Table 2.

### 3.5. Publication Bias

Egger's and Begg's tests revealed that there was no publication bias (Table 2).

### 3.6. Sensitivity Analysis

Sensitivity analysis adopts a one-by-one elimination method. The results showed that the combined effect size of each index did not dramatically change, and the results did not reverse. Therefore, the results of this meta-analysis are stable.

## 4. Discussion

ctDNA is one of the main components of liquid biopsy, which can reflect the intrinsic molecular characteristics of tumours and can monitor the dynamic changes in the tumour genome in real time. This has important clinical guiding significance for individualized clinical medication [31–33]. ctDNA was first found in the blood of healthy people, but it was not seriously considered [34]. In 1977, Leon et al. [35] found that the concentration of ctNDA was elevated in patients with lymphoma and tumours of the lung, ovary, uterus, and cervix. With the development of detection technologies, such as ddPCR and ARMS PCR, ctDNA detection has become more accurate, efficient, and noninvasive and is expected to become a potential tumour prognostic biomarker. The concentration of ctDNA in patients is related to the type, stage, and progression of tumours. The plasma ctDNA concentration of localized cancer patients was lower than that of metastatic cancer patients, and the concentration of mutant DNA fragments was relatively higher in advanced cancer patients or metastatic patients [36, 37]. The concentration of ctDNA in patients with metastatic cancer is higher than most commonly used biomarkers, and there is a similar relationship between patients with advanced breast cancer and the concentration of ctDNA [38]. Lee et al. [39] found that ctDNA mutation status can predict the recurrence of breast cancer and adverse survival outcomes. Chen et al. [40] found that a higher ctDNA concentration is related to the poor survival rate of pancreatic cancer. However, the relationship between ctDNA and HCC is unclear. Therefore, it is necessary to comprehensively analyse the clinical application of ctDNA in the prognosis prediction of HCC patients.

To the best of our knowledge, this is the first systematic review to explore the relationship between ctDNA status and prognosis in HCC patients. Our meta-analysis found that ctDNA is related to the deterioration of DFS/OS in HCC patients. Due to the limited number of included studies, the DFS results need to be interpreted with caution. Due to the limited information provided by the included literature, further analysis of pathological characteristics is not possible. ctDNA detection is also related to tumour size and TNM staging, which can be explained by the theory that circulating tumour ctNDA is related to the patient's tumour burden and aggressiveness [41].

The occurrence of liver cancer is a complex multistep process in which many signal cascades are altered, resulting in a different molecular profile [42]. The main mutations include TP53, SOCS3, CTNNB1, and TERT mutations. Mutations in the TERT promoter are found in approximately 50% of HCC, are the most common somatic genetic changes in HCC, and are involved in the early stages of HCC [43, 44]. In solid tumours, such as lung cancer and breast cancer, the presence of mutations in the TERT promoter is closely related to poor prognosis [45]. Our subgroup analysis found that TERT is related to the poor prognosis of HCC. SOCS3 can inhibit the activation of STAT3 and the expression of downstream target genes, thereby preventing the malignant transformation of cells and promoting cell apoptosis [46, 47]. Abnormal methylation of the SOCS3 promoter is involved in the occurrence and development of HCC and is related to the poor prognosis of HCC [48]. Our subgroup analysis found that only SOCS3 promoter mutations were related to DFS. Due to the limited literature on subgroup analysis based on the markers in ctDNA, more studies are needed for verification.

In addition, the clinical stage of HCC is closely related to the choice of prognosis and treatment. Barcelona clinical liver cancer (BCLC) stage and Hong Kong liver cancer (HKLC) stage are commonly used clinical staging systems [49, 50]. The HKLC staging system is based on hepatitis B virus infection, which is suitable for most Asian countries [50]. Relevant studies have shown that HKLC has more detailed staging and corresponding treatment strategies than BCLC and also provides considerable value for the prognosis of patients [51, 52]. Studies [23] have shown that ctDNA is directly related to BCLC stage, and it is therefore suggested to combine ctDNA with the HCC staging system to predict the prognosis of HCC patients. As the population included in this study was all Asian, HKLC may be more suitable for combining with ctDNA to determine the prognosis of HCC patients. The new scoring model constructed by supplementing the traditional scoring model with more sensitive and accurate novel markers will certainly better guide the subsequent treatment of HCC in the future.

Several limitations in this meta-analysis should be addressed. First, the lack of widely accepted ctDNA gene targets in HCC patients may be one reason for the deviation. HCC is considered to be a malignant tumour with high histological and aetiological heterogeneity. Therefore, customizing more circulating genes according to the latest molecular characteristics will help the detection of ctDNA and its clinical application in HCC. Due to the nature of our research, with the abundance of studies reporting positive results, selection bias may appear. In addition, the detection methods and materials were different, even if we conducted a subgroup analysis. Due to the limited number of included studies, this may have also caused research bias. We also did not have enough data to compare the changes in ctDNA before and after surgery, which limits the clinical application of ctDNA. Finally, the included studies were from Asia, and our conclusions may not be universally applicable.

## 5. Conclusions

Despite the above limitations, this study is still the first meta-analysis to analyse the relationship between ctDNA and the prognosis of HCC patients from both quantitative and qualitative aspects. Our research shows that ctDNA positivity is significantly related to the poor prognosis of HCC patients. ctDNA is an effective marker for evaluating the prognosis of HCC and can provide more effective information for HCC treatment decisions. In addition, mutations in the TERT and SOCS3 promoters in ctDNA are associated with poor prognosis and can be used as good targets for liquid biopsy to help select treatment strategies. Despite this, multicentre, prospective large-scale clinical studies are still needed to further verify this conclusion and provide a more scientific basis for promoting the clinical application of liquid biopsy technology in HCC.

## Figures and Tables

**Table 1:** General characteristics of the study populations.

First author and publication year	Country	Ethnicity	No. of patients	TNM stage	Sample origin	Time of sample collected	Detection methods	Treatment	Marker	Endpoints	Survival data type			NOS score
											Survivorship curve	Univariate	Multivariate	
Hirai 2020 [30]	Japan	Asian	130	II–IV	Plasma	Posttreatment	ddPCR	Chemotherapy	TERT	OS	2.34 (1.46–4.02)	2.19 (1.36–3.61)	1.94 (1.18–3.24)	8
Wang 2020 [23]	China	Asian	81	I–IV	Plasma	Pretreatment	ddPCR	Surgery	ctDNA	OS	6.82 (2.67–17.42)	6.52 (2.52–16.87)	4.20 (1.50–11.80)	8
										DFS	5.46 (2.49–12.56)	NA	NA	
Ako 2020 [28]	Japan	Asian	36	I–III	Serum	Pretreatment	ddPCR	Surgery	TERT	DFS	NA	3.28 (1.23–11.3)	3.01 (1.11–10.5)	7
Wei 2018 [25]	China	Asian	119	NA	Plasma	Pretreatment	MSP	Surgery	SOCS3	OS	1.12 (0.98–2.44)	NA	NA	7
										DFS	2.64 (1.14–3.48)	NA	NA	
Huang 2011 [24]	China	Asian	72	I–IV	Plasma	Pretreatment	Real-time PCR	Surgery	ctDNA	OS	1.34 (0.96–2.34)	NA	NA	7
Rem 2006 [22]	China	Asian	79	I–IV	Plasma	Pretreatment	Ultraviolet transilluminator	Surgery	ctDNA	OS	2.46 (1.26–4.56)	NA	NA	7
										DFS	1.96 (1.32–3.23)	NA	NA	
An 2019 [29]	China	Asian	26	I–IV	Plasma	Pretreatment	Qubit fluorometer	Surgery	ctDNA	DFS	3.16 (1.46–6.42)	NA	NA	7
Cai 2019 [21]	China	Asian	34	I–IV	Plasma	Pretreatment	Illumina sequencing	Surgery + other adjuvant therapies	ctDNA	OS	11.86 (2.84–47.51)	12.08 (2.92–46.25)	10.69 (2.84–45.81)	8

ddPCR, droplet digital PCR; DFS, disease-free survival; OS, overall survival; NA, data not applicable.

**Table 2:** Results of subgroup analysis.

Survival	Variables	Parameters	*n*	HR (95% CI)	*P* value	Heterogeneity		Publication bias	
						*I* ^2^ (%)	*P*_*h*_	Begg's *P*	Egger's *P*
Survivorship curve OS	Overall	—	6	2.44 (1.42, 4.20)	**0.001**	77.6	**<0.001**	1.000	0.486
	Method	ddPCR	2	3.70 (1.31, 10.45)	**0.013**	74.2	0.049		
		Others	4	1.97 (1.05, 3.70)	**0.035**	75.7	0.006		
	Marker	TERT	1	2.34 (1.41, 3.88)	**0.001**	—	—		
		ctDNA	4	3.46 (1.43, 8.34)	**0.006**	81.2	0.001		
		SOCS3	1	1.12 (0.71, 1.75)	0.626	—	—		
Univariate OS	Overall	—	3	4.48 (1.17, 13.70)	**0.003**	75.1	0.018	0.602	0.532
	Method	ddPCR	2	3.49 (1.21, 10.05)	**0.021**	75	0.045		
		Others	1	12.08 (3.04, 48.08)	**<0.001**	—	—		
	Marker	TERT	1	2.19 (1.34, 3.57)	**0.002**	—	—		
		ctDNA	2	7.95 (3.63, 17.40)	**<0.001**	0	0.471		
		SOCS3	0	—	—	—	—		
Multivariate OS	Overall	—	3	3.74 (1.45, 9.65)	**0.006**	67.2	0.047	0.117	0.242
	Method	ddPCR	2	2.49 (1.23, 5.06)	**0.012**	42.5	0.187		
		Others	1	10.69 (2.84, 45.81)	**0.001**	—	—		
	Marker	TERT	1	1.94 (1.17, 3.21)	**0.010**	—	—		
		ctDNA	2	5.94 (2.45, 14.37)	**<0.001**	10.6	0.290		
		SOCS3	0	—	—	—	—		
Survivorship curve DFS	Overall	—	4	2.63 (1.96, 3.53)	**<0.001**	40.3	0.170	0.624	0.302
	Method	ddPCR	1	5.46 (2.49, 12.56)	**<0.001**	-	-		
		Others	3	2.35 (1.71, 3.22)	**<0.001**	0	0.493		
	Marker	TERT	0	—	—	—	—		
		ctDNA	3	2.62 (1.86, 3.71)	**<0.001**	60.2	0.081		
		SOCS3	1	2.64 (1.51, 4.61)	**0.001**	—	—		
Univariate DFS	Overall	—	1	3.28 (1.23, 11.30)	**0.011**	—	—	—	—
Multivariate DFS	Overall	—	1	3.01 (1.11, 10.5)	**<0.001**	—	—	—	—

ddPCR: droplet digital PCR; DFS: disease-free survival; OS: overall survival; *P* value = 0.05 was considered statistically significant; CI: confidence interval; HR: hazard ratio. The bold parts indicate statistical differences.

## Data Availability

The data used to support the findings of this study are available from the corresponding author upon request.
